# Understanding mitochondrial potassium channels: 33 years after discovery

**DOI:** 10.3389/abp.2024.13126

**Published:** 2024-05-28

**Authors:** Adam Szewczyk

**Affiliations:** Laboratory of Intracellular Ion Channels, Nencki Institute of Experimental Biology, Polish Academy of Sciences, Warsaw, Poland

**Keywords:** mitochondria, potassium channels, reactive oxygen species, cytoprotection, potassium channel openers

## Abstract

Mitochondrial investigations have extended beyond their traditional functions, covering areas such as ATP synthesis and metabolism. Mitochondria are now implicated in new functional areas such as cytoprotection, cellular senescence, tumor function and inflammation. The basis of these new areas still relies on fundamental biochemical/biophysical mitochondrial functions such as synthesis of reactive oxygen species, mitochondrial membrane potential, and the integrity of the inner mitochondrial membrane i.e., the passage of various molecules through the mitochondrial membranes. In this view transport of potassium cations, known as the potassium cycle, plays an important role. It is believed that K^+^ influx is mediated by various potassium channels present in the inner mitochondrial membrane. In this article, we present an overview of the key findings and characteristics of mitochondrial potassium channels derived from research of many groups conducted over the past 33 years. We propose a list of six fundamental observations and most important ideas dealing with mitochondrial potassium channels. We also discuss the contemporary challenges and future prospects associated with research on mitochondrial potassium channels.

## Introduction

**FIGURE 1:**
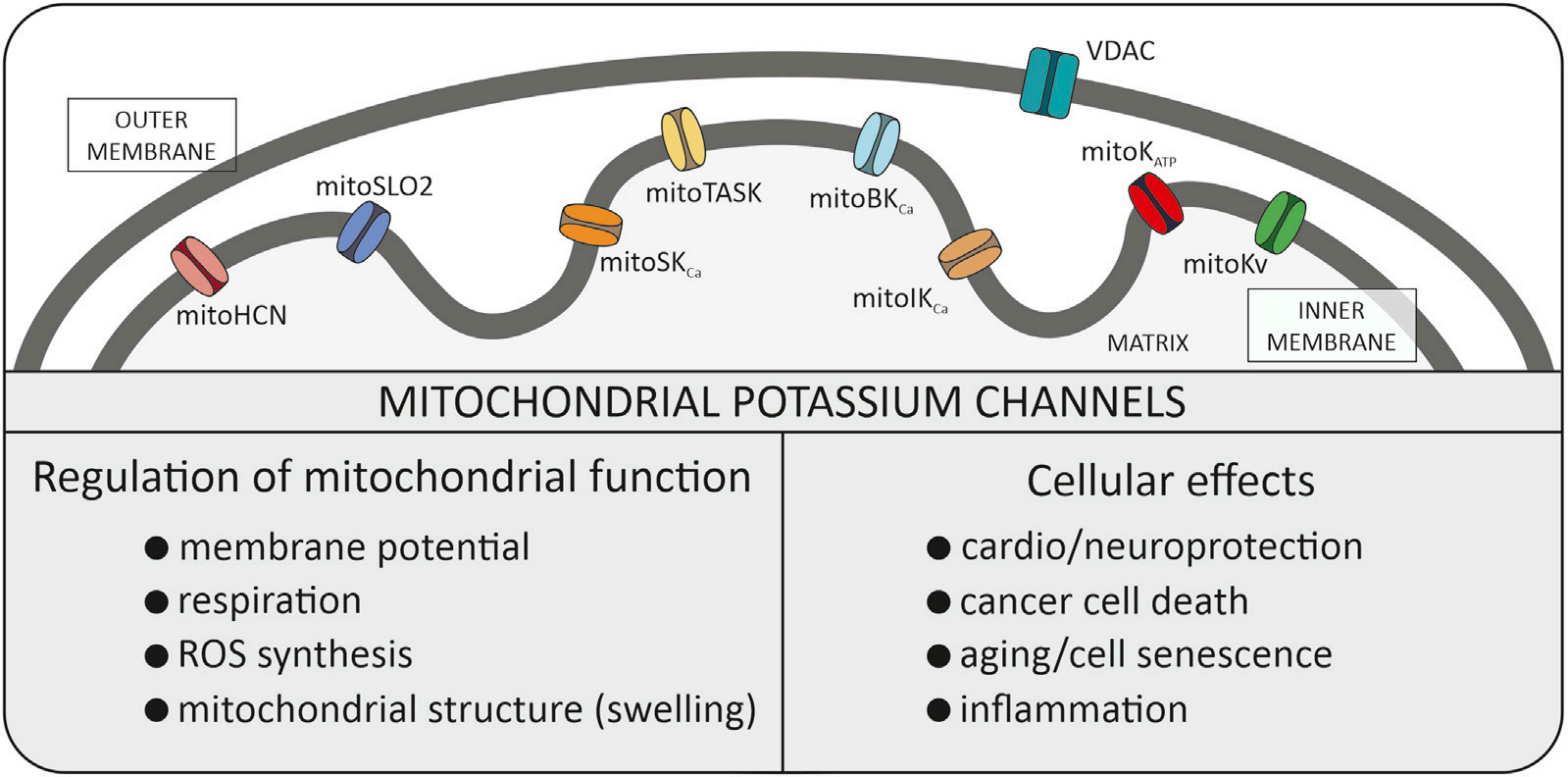
Potassium channels identified in the inner mitochondrial membrane. All these types of channels were described in the manuscript. Additionally, the biophysical role in mitochondria and physiological role within the cell is summarized. VDAC, voltage dependent anion channel (porin).

When investigating the fundamentals of mitochondrial function within cells, we can identify several simple cations that form the basis of many processes (Szabo and Zoratti, 2014). It is well known that the proton gradient serves as the driving force for ATP synthesis in mitochondria. The Ca^2+^ cations entering the mitochondria not only buffer the cytosolic pool of these ions but can also contribute to some physiological situations such as the mitochondrial mega-channel activation (Carraro and Bernardi, 2023; Zoratti et al., 2024). The effects of Mg^2+^ on mitochondrial functions such as energy metabolism, mitochondrial Ca^2+^ handling, and apoptosis are well established (Liu and Dudley, 2020). Mitochondrial Na^+^ have been discovered as a new second messenger regulating inner mitochondrial membrane (IMM) fluidity and reactive oxygen species (ROS) generation by respiratory chain complex III (Hernansanz-Agustín and Enríquez, 2022). In this study, we will focus on the properties and the role of K^+^ transport, via potassium channels (mitoK channels) present in IMM (Szewczyk, 1996; Kicinska et al., 2000; Debska et al., 2001; O’Rourke, 2007; Singh et al., 2012; Szabo and Szewczyk, 2023) (Figure 1).

In general, proper mitochondrial function is based on the integrity of mitochondrial membranes. Peter Mitchell in his Nobel Lecture delivered in 1978 underlined the importance of the low permeability of the IMM to ions (Mitchell, 1985). Consequently, the discovery of multiple potassium channels in the IMM was for many years considered to be an experimental artifact. Nowadays, mitoK channels present in the IMM are recognized as crucial players for regulating some mitochondrial function (Kravenska et al., 2021; Szabo and Szewczyk, 2023). The mitoK channels have attracted attention for many years, especially in the context of the regulation of life/death processes in the various cell types (Garlid, 2000). For example, the activation of mitoK channels may induce cytoprotective phenomena in cardiac tissue and in neurons (O’Rourke, 2007). On the contrary, inhibition of mitoK channels may cause cell death (Checchetto et al., 2021).

In this paper, we will present what we consider to be the most significant discoveries/ideas in the field of mitoK channels over the past 33 years. These subjective, proposed by author, list of six the most important observations are as follows: 1). Discovery of mitoK channels in various tissues and identification of their molecular identity; 2). Cytoprotection (cardioprotection, neuroprotection) induced by mitoK channels activation; 3). Cancer cell death by mitoK channels inhibition; 4). Role of mitoK channels in aging/senescence/life span; 5). Interactions of mitoK channels with respiratory chain; 6). Druggability of the mitoK channels.

## Mitochondrial potassium channel discovery

In 1991, an ion channel selective for K^+^ was discovered in the IMM of rat liver mitochondria (Inoue et al., 1991), confirming previous findings on channels in mitochondria by Catia Sorgato (Sorgato et al., 1987). However, what significantly altered the interpretation of this experimental data was the revelation that the channel exhibited not only K^+^ selectivity but also susceptibility to inhibition by ATP and the antidiabetic sulfonylurea, glibenclamide (Inoue et al., 1991). This discovery situated mitoK channels within a similar family to ATP-regulated potassium channels found in the plasma membrane of pancreatic beta-cells, cardiomyocytes, neurons, and others (Szewczyk, 1996; O’Rourke, 2007). Undoubtedly, this observation served as a pivotal starting point for numerous experiments identifying ATP-regulated mitochondrial potassium (mitoK_ATP_) channels across various tissues, notably cardiomyocytes (Szewczyk et al., 2009; Szabo and Szewczyk, 2023). Following several years of intensive investigation across multiple laboratories into the functional role of these channels, it was demonstrated that the activation of mitoK_ATP_ channels (by potassium channel openers) induces a cardioprotective phenomenon (Liu et al., 1999; Garlid, 2000; Szteyn and Singh, 2020; Lukowski et al., 2022). Subsequently, similar findings in neural tissue suggested the involvement of these channels in neuroprotection (Busija et al., 2004; Bednarczyk, 2009). In summary, the association of mitoK_ATP_ channels with cytoprotection marked a significant milestone in the rapid development of the mitochondrial potassium channel field. Moreover, other mitoK channels (such as large conductance calcium-activated potassium—mitoBK_Ca_ channels) were later implicated in cytoprotection across various cell types (Xu et al., 2002). Despite a plethora of observations, however, the biochemical mechanisms underlying these events remain unclear. It is probable that the indirect modulation of ROS generation by mitoK channels (via depolarization of mitochondria) contributes to this phenomenon.

In recent years, researchers have demonstrated that the family of mitoK channels constitutes one of the most numerous classes of mitochondrial channel proteins. They are also present in plants and in simple organisms (Koszela-Piotrowska et al., 2009; Matkovic et al., 2011; Laskowski et al., 2015). It includes not only mitoK_ATP_ and mitoBK_Ca_ channels but also intermediate conductance (mitoIK_Ca_), and small conductance (mitoSK_Ca_), voltage-regulated potassium (mitoKv1.3, mitoKv7.4) channels, mitochondrial hyperpolarization-activated cyclic nucleotide-gated (mitoHCN) channels, mitochondrial sodium-activated potassium (mitoSlo2) channel and two-pore domain potassium (mitoTASK-3) channel (Szabo and Szewczyk, 2023). The activity of potassium channels are regulated by various stimuli, such as pH, Ca^2+^ and ROS (Szabo and Szewczyk, 2023). The mitoK channels have been identified in many tissues but at the same time their number of molecules in mitochondrial membranes is relatively small compared to other mitochondrial transport proteins. Probably low density of mitoK channels and channel run down phenomenon were reasons for questioning in the 90’s the presence of these channels at all.

Another issue regarding mitoK channels is the following: why is such a simple process, like K^+^ influx into a matrix, facilitated by a wide variety of potassium channels? For example, in cardiac mitochondria, six mitoK channels have been identified: mitoK_ATP_, mitoBK_Ca_, mitoSK_Ca_, mitoSlo2, mitoHCN channels and mitoKv7.4 channels (Szabo and Szewczyk, 2023). What is the physiological benefit of using many different ligands and factors to regulate these channels? Probably, potassium channels present in cardiomyocyte mitochondria are activated under specific physiological circumstances (Kulawiak et al., 2021). An early event during cardiac ischemia is ATP depletion. This is followed by mitochondrial membrane depolarization. Moreover, because of ATP depletion, ion pumps cannot function, leading to an increase in the cellular Ca^2+^ concentration. The rise in Ca^2+^ during ischemia and reperfusion leads to an overload of mitochondrial Ca^2+^, during reperfusion when oxygen is reintroduced. The decrease in intracellular pH during severe ischemia promotes the imbalance of other cations and leading to cellular Na^+^ overload (Kulawiak et al., 2021). These complex changes may lead to channel activation/inhibition possibly explains why there are few potassium channels in cardiac mitochondria. Most likely, the timing of ATP, pH, Ca^2+^, and Na^+^ concentration changes is critical to control K^+^ flux in mitochondria stabilizing structure of mitochondria.

Molecular identity of mitoK channels for many years was a mystery. Lack of molecular mitoK identity was an argument questioning the presence of potassium channels in mitochondria. Let’s summarize this long way of channel molecular identity recognition. Today we believe that mitoBK_Ca_ channel is one of the splice variants of KCNMA1 (Slo1) gene (Singh et al., 2013; Galecka et al., 2021). Properties of mitoBK_Ca_ suggest that the pore-forming subunit is encoded by the same gene coding for plasma membrane BK_Ca_. Several studies suggested that the VEDEC BK_Ca_ isoform is located in the IMM. With the mitoK_ATP_ channel there is a more complex situation. It can not be excluded that K^+^ influx is catalyzed by 2-3 various proteins in various tissues. Recently, it was shown that the pore-forming subunit of the mitoK_ATP_ channel is a product of the CCDC51 gene (Paggio et al., 2019). The mitoK_ATP_ is inhibited by the antidiabetic sulfonylurea glibenclamide. Therefore, it was speculated that the glibenclamide receptor (product of ABC8/MITOSUR gene) is an integral part of the mitoK channel. Indeed the mitoK_ATP_ channel formed by these two proteins has the established pharmacological properties of the mitoK_ATP_ channel (Paggio et al., 2019). Previous studies showed that also the ROMK2 potassium channel isoform of the renal outer medullary potassium channel could be the component of the mitoK_ATP_ channel (Bednarczyk et al., 2018; Laskowski et al., 2019). Detailed discussion on mitochondrial potassium channel molecular identity was recently reviewed (Szabo and Szewczyk, 2023).

The presence of various auxiliary β subunits in mitoBK_Ca_ channels and sulfonylurea receptors in the mitoK_ATP_ channel causes that, despite undoubted progress in the identification of channel proteins, the problem of their detailed identification is still a challenge for the future (Piwonska et al., 2008).

## From cytoprotection to cell death

The mitoK channels have been described as an important player in cellular pro-life and death signaling. The activation of mitoK channels (by potassium channel openers), such as ATP-regulated or calcium-activated large conductance potassium channels, may have cytoprotective effects in cardiac or neuronal tissue (Liu et al., 1999; Busija et al., 2004). This concept was a strong driving force of studies in many laboratories. Potassium channel opener induced cytoprotection is also induced by endogenous signaling via protein kinases (Frankenreiter et al., 2017).

It has also been shown that inhibition with channel blockers of the mitochondrial Kv1.3 channel may lead to pancreatic cancer cell death (Leanza et al., 2014). But still there is an open question to what extent mitoK channels are promising drug targets in various organs and tissues? Future prospects of the druggability concept of mitoK channels was evaluated recently (Wrzosek et al., 2020).

## Searching for new functions of mitochondrial potassium channels

The putative functional roles of these channels involve alterations in mitochondrial matrix volume, mitochondrial respiration, and protonmotive force (membrane potential) (Czyz et al., 1995). Furthermore, the activity of these channels influences the generation of ROS by mitochondria (Kulawiak et al., 2008; Kulawiak et al., 2023). The activity of mitochondrial potassium channels is subject to modulation by various intrinsic signals, including Ca^2+^ concentration, membrane potential, phosphorylation, and membrane stretching (Szabo and Szewczyk, 2023).

It was demonstrated that BK_Ca_ channels are present in *Drosophila melanogaster* mitochondria, and channel mutants induce structural and functional defects in mitochondria leading to an increase in ROS (Gururaja Rao et al., 2019). It was found that the absence of BK_Ca_ channels reduced the lifespan of *Drosophila*, and overexpression of human BK_Ca_ channels in flies extends their life. This suggested a potential role of mitoK channels and ROS in regulating mitochondrial functional integrity, and lifespan (Gururaja Rao et al., 2019). Probably mitoBK_Ca_ play a role in cellular senescence induced by oxidative stress (Gluchowska et al., 2023).

## Mitochondrial context of potassium channel regulation

The mitochondrial respiratory chain comprises a series of complex organized redox reactions generating a protonmotive force and, consequently, ATP synthesis. Certain redox centers, such as complexes I and III of the mitochondrial respiratory chain are sources of ROS. Mitochondrial generated ROS can influence remotely the activity of mitoK channels. But there are some indications proposing an alternative, a direct mechanism for the regulation of mitoK channels by the respiratory chain.

It is well-known that mitoK channels interact with various mitochondrial proteins, some of which are involved in the respiratory chain. These observations were recently summarized (Lewandowska et al., 2024). For instance, it has been suggested that mitoK_ATP_ channels interact with succinate dehydrogenase. In cardiac mitochondria, it was found that the β1 subunit of the mitoBK_Ca_ channels interacts with Cytochrome c Oxidase (COX) subunit I. Furthermore, studies have demonstrated that other respiratory chain protein complexes interact with mitoBK_Ca_ channels in both cardiac and brain mitochondria. Additionally, mitochondrial tandem pore domain K^+^ channels TASK-3 interact also with the respiratory chain. A recent report revealed a similar interaction between the mitoKv1.3 channel and respiratory chain complex I (for review see Lewandowska et al., 2024).

We found that the activity of mitoBK_Ca_ channels in glioblastoma cells is regulated by substrates and inhibitors of the respiratory chain (Bednarczyk et al., 2013). This study suggested that COX is a key element of this kind of channel regulation (Bednarczyk et al., 2013). Moreover, given that COX is the primary infrared-absorbing protein, it raises questions about the potential light regulation of mitoK channels (Szewczyk and Bednarczyk, 2018).

Further research will be important to clarify the functional consequences of these interactions. Undoubtedly, this form of regulation may prove to be unique for mitoK channels. The exact nature and functional implications of these interactions remain unclear. This kind of direct functional coupling between the energy generating system (respiratory chain) with the energy dissipation system (potassium channels) may lead to an interesting putative regulatory mechanism in mitochondria.

Recently other functional/structural coupling within the mitochondrial potassium channel was observed. It was found that mitochondrial potassium channel ROMK2 may interact with two lipid kinases: acylglycerol kinase (AGK) and diacylglycerol kinase ε (DGKE), which are localized in mitochondria (Krajewska et al., 2024). Additionally, it was found that the products of AGK and DGKE, lysophosphatidic acid (LPA) and phosphatidic acid (PA), stimulated the activity of ROMK2 potassium channels reconstituted in planar lipid bilayers (Krajewska et al., 2024).

The structure/function interplay of mitoK channels alongside other mitochondrial proteins suggests a new dimension in mitoK channels regulation. The exceptionally high membrane potential of the IMM and its potential for ROS generation may characterize significant signaling pathways within cells.

## The troublesome pharmacology of mitochondrial potassium channels

In order to influence activity of various mitoK channels, numerous research groups continually explore novel compounds hoping to find molecules with high specificity for mitoK channels (Szewczyk and Marban, 1999; Augustynek et al., 2017; Leanza et al., 2019). The existing literature already reports positive protective effects on ischemia/reperfusion processes through the activation of mitoK_ATP_ channels by the potassium channel opener - diazoxide, and the mitoBK_Ca_ channels by potassium channel opener NS1619 and its follower NS11021 (Szewczyk et al., 2006). Nevertheless, it is noteworthy that these compounds exhibit limited specificity towards mitoK channels. Application of these substances in the micromolar concentration range unmasks a variety of side effects (Wrzosek et al., 2022). It is important to remember that molecules with some hydrophobicity of positive charge (in physiological pH) will be accumulated by mitochondria. It is due to very high membrane potential (up to—180 mV) on the IMM, with negative polarization of the mitochondrial matrix. For example, a 10 nM drug present in cytosol could accumulate up to 10 µM concentration in a matrix (Kowaltowski and Adbulkader, 2024). At this concentration range the probability of nonspecific interaction with some of ∼1,500 mitochondrial proteins is very high. In contrast, toxins isolated from the venom of various scorpion species such as iberiotoxin specifically (at low concentration) inhibit the activity of mitoBK_Ca_ channels (Augustynek et al., 2017). But application of this peptide to block mitoBK_Ca_ channels on intact cells is practically impossible.

Developing very selective channel blockers and potassium channel openers targeting mitoK channels is a significant challenge in this field. Recently it was shown that selective targeting of mitoIK_Ca_ channel (Bachmann et al., 2022), mitoTASK channel (Bachmann et al., 2021) and mitoKv channel (Severin et al., 2022) is possible.

## Discussion

Over the past 33 years since the identification of the first potassium channel in the IMM, research in this field has made significant progress (Kulawiak and Szewczyk, 2022). This pathway started from identification of the mitoK channels that met with skepticism by the bioenergetics community to current research placing these channels in the phenomena of cytoprotection, cellular senescence, and neoplastic cell death. What limits further development of this field?

First, access to good pharmacology is the “dark side” of this field (Szewczyk et al., 2010; Olszewska and Szewczyk, 2013; Leanza et al., 2019). Because mitoK channels are similar to those located in plasma membranes, it is very difficult to identify pharmacological modulators specific only for mitoK channels (Szewczyk and Wojtczak, 2002; Citi et al., 2018). The unique high membrane potential of mitochondria may help to discriminate targeting of some drugs to mitoK channels (Testai et al., 2015; Wrzosek et al., 2020).

The second limiting factor for further progress is the development of new techniques to measure channel activity *in situ*, that is, within an intact cell. Majority of techniques currently applied in the studies are based on cell fractionation and mitochondria isolation (Walewska et al., 2022). By definition in this process we lose a network of signaling pathways where mitoK channels are potentially involved (Walewska et al., 2018). Probably progress in synthesis of potassium specific fluorescent probes may solve this problem. Unfortunately, there are other potassium transport proteins in mitochondria.

The third challenge for the future involves further identifying the molecular identity of various mitoK channels. This aim will not only expand our understanding of the system but also will start new avenues of research, such as *in vitro* translation with lipid nanodiscs and the application of various biophysical techniques. Additionally, it will aid in the identification of protein neighborhoods, clarification of the import machinery, and more.

In summary, mitoK channels, considered the “younger siblings” of the potassium channels found in plasma membranes, play a crucial role in some cellular signaling pathways. The mitoK location within mitochondria, which serve as hubs for fundamental metabolic and signaling functions, highlight their significance. The author believes that the future of this field holds exciting prospects.
